# “You Want Them Pretty, but Not Too Intelligent!”: Everyday Talk and the Continuum of Men's Violence Against Women in Forensic Institutional Care

**DOI:** 10.3389/fpsyt.2022.886444

**Published:** 2022-06-06

**Authors:** Emma C. Joyes, Mel Jordan

**Affiliations:** ^1^Population Health Sciences Institute, Faculty of Medical Sciences, Newcastle University, Newcastle upon Tyne, United Kingdom; ^2^School of Sociology and Social Policy, University of Nottingham, Nottingham, United Kingdom

**Keywords:** Violence Against Women and Girls (VAWG), forensic mental health, sexual offending, sexual violence, rehabilitation, hegemonic masculinity, misogyny, ethnography

## Abstract

The forensic setting houses persons with offence convictions who are also in receipt of ongoing mental healthcare–a criminal justice system and healthcare meeting-point. Extant literature highlights how this context is laden with interpersonal and institutional difficulties unique to a secure context that must provide care and custody concurrently. Our central argument is that the intertwining and interdependent cultural and custodial elements of forensic healthcare environments are integral and influential to care, culture, and conduct within such institutions–including concerning misogynistic everyday talk and the continuum of men's violence against women therein. We argue that the institution is a continuation of contemporary social issues experienced within community life (e.g., misogyny), as the boundaries of such institutions are porous–polis values traverse physical brickwork. This paper analyses ethnographic data from two male wards that are situated within a UK inpatient forensic mental health hospital. Ethnographic fieldwork occurred over 300 hours–overtly participating in, exploring, and recording the daily life of the community. Five excerpts of ethnographic data are presented, which evidence the gendered ward environment and highlight a series of encounters pertaining to problematic social life, which are the upholding of heteronormative gender roles, hegemonic masculinity, and misogyny. These views are problematised within the sexual offending rehabilitative context by considering the clinical risk associated. Further, we argue that to only focus on the end of the continuum often viewed as most serious (e.g., rape) ignores a pervasive cultural landscape of the polis in wider community, beyond the institution, that facilitates the more commonly experienced end of the continuum related to misogynistic values, encounters, and talk. We evidence how social norms and habitualised gendered actions permeate the institution, which bring into question the rehabilitative efficacy of the hospital. This paper embraces a feminist lens to explore everyday social interactions and the embodied experience of the female ethnographer within a male-dominated forensic setting. We contribute to the literature by newly theorising the influences of hierarchical heterosexual gender roles, violent language in forensic settings, and misogynistic attitudes and practice, on the care for, and rehabilitation of, patients.

## Introduction

The forensic mental health context has been described as unintentionally toxic (1) and the environment has been described as “a particularly volatile place to live” [(2), p. 2581]. Inpatient care within forensic institutions is fraught with challenges for those in receipt of care and for those who provide support to such individuals. Caregivers within forensic psychiatric institutions must perform care and custody concurrently, wherein role conflict occurs and professional and personal values are challenging–institutional work is emotional work (3). The complex and sometimes toxic social environment can sharply contrast the positive and supportive therapeutic relationships that are integral to care practice which promote recovery. The complex social environment has been more thoroughly discussed within an earlier publication (4), however this paper focusses on misogynistic attitudes which are linked to Violence Against Women and Girls (VAWG) evidenced within the ethnographic data. The normalisation of values that subjugate women in a workplace setting can influence a setting's staff; this is doubly pertinent in secure services, as they're argued to instil emotional isolation from family, friends, and colleagues (3). For the ethnographer in this study, the everyday talk, and the continuum of men's violence against women in forensic institutional care, made for an uncomfortable period of fieldwork.

### Interpersonal Recovery: The Value of Social Relationships

The notion that individuals do not recover in isolation (5–7) and that relationships are central to recovery is supported within this paper. Within mental healthcare, professionals are encouraged to build reciprocal relationships with patients, with mutuality underlying many models and theories of nursing care (8). The quality of therapeutic relationships has been noted to be important for promoting recovery (9, 10). Relationships have been argued to not just be an important part of a mental health intervention–“they *are* the intervention” (11) and the introduction and the nurturing of relationships hold an important therapeutic value (12, 13). Forensic mental healthcare increasingly adopts a multidisciplinary approach to teamwork, wherein staff collaborate “working to the same end, namely the successful treatment and rehabilitation of the patient” [(14), p. 104]. Within forensic mental health nursing McMurran et al. [(14), p. 96] highlight the (Department of Health supported) importance of values within daily practice, inclusive communications, psychosocial care (including social networks and relationships), and personal development, plus “a respectful attitude to the patients in their care.” Secure setting work combines custody, therapy, and the culture of the setting.

Campling et al. (15) argue we are all constructions of our environment and of each other, developing our identities, learning patterns of communicating, and our social responses in the context of our social environment. Whilst formal therapy is integral to mental healthcare, the social environment and interactions—the everyday encounters—hold an important therapeutic value, contributing to the therapeutic milieu (16). Research within the forensic mental health context supports the notion that everyday encounters can be therapeutic, where staff who engage in relational small talk with the aim to socially connect with those in receipt of care is valued (12). However, such connexion can be facilitated by “lads talk” [(17), p. 177]. Whilst this may promote intra-group relations for males, such social encounters may also serve to support the oppression of women, depending on the content of the conversation.

### The Staff-Patient Relationship: Paternalism

The relationship between staff and individuals in receipt of care can be challenging, particularly within the forensic context (18, 19), and institutional and professional constraints within mental healthcare can limit the potential for mutuality (8). Staff are required to navigate a dual role, one of carer and one of custodian (20–22), with staff adopting both a “relational” and a “parentalistic and behaviour-changing” approach to care [(23), p. 359]. The paternal or parental model to care adopts a corrective approach where staff are viewed as promoting socially acceptable behaviour (24, 25). The behaviour of the patient then becomes the focus of care (23). Staff are conceptualised, in theory and frontline practice, as models for apt behaviour-this is an important recognition for the analysis section which follows in this paper.

This paternalistic model is underpinned by a disparity between staff (who are deemed well) and those who are in receipt of care (who are deemed unwell) where the role of helper and helpless is commonly reinforced in care (26). Providing care within a custodial context is challenging (27)-wherein it's integral to care that building and maintaining relationships occurs, however the environment is highly emotive (4). It is a long-standing position in medical sociology that medical knowledge “is socially contingent. It is argued that medical knowledge is socially constructed” [(28), p. 13]. Criticism of biomedicine and the dominance of clinical knowledge is well-rehearsed. What's relevant here is that social relations can be mediated by medical knowledge (29) and that medical knowledge is controlled by those who manage its means of production (30). This reiterates the importance of culture–clinical culture in this instance–within care settings.

### Rehabilitative Approaches for Sexual Offences

The Good Lives Model (GLM) is the dominant approach to offender rehabilitation within the UK, Canada, Australia, and New Zealand, which is underpinned by a risk-need theoretical approach (31). The GLM is known as a strengths based model and promotes the development of a self-determined life (32, 33). Sexual offending, has been argued to reflect “socially unacceptable and often personally frustrating attempts to pursue primary human goods” [(34), p. 90]. According to the GLM, sexual violence can be the result of one of two primary goods, mastery or relatedness. Mastery may be pursued in order to gain power over an individual (35). A risk factor outlined in the Structured Assessment of Risks and Needs (SARN) risk assessment tool in which“[a] view of heterosexual relationships where the male is seen as dominant and the female as submissive” is seen as problematic [(36), p. 103]. Thus, clinical assessment tools support the notion that males who view women as submissive and engage in behaviour in which they strive to gain relational power over women is erroneous and laden with risk. Relatedness as a motivation for sexual offending proposes that the individual is aiming to achieve intimacy, but the utilisation of controlling behaviour is unlikely to lead to a satisfying level of intimacy (37, 38). However, the notion that the individual is aiming to achieve intimacy through sexual violence is debated within the feminist literature, with continuing arguments concerning “whether rape is about sex or whether it is about violence or power” [(39), p. 31]. The history and contemporary issues relating to these debates have been explored more thoroughly elsewhere [for example, see: (39–42)]. It is important to note that the motivations for rape as power (mastery) or sex (intimacy) underpin clinical rehabilitative work (e.g., the GLM and SARN risk assessment tool).

Walton and Hocken (43) highlight how third wave interventions with persons with sexual convictions evidence the importance of “thinking and language” (p. 154). Walton and Hocken (43) review Acceptance and Commitment Therapy and demonstrate the importance of value-consistent living, here-and-now (not past) attention, and coaching-style/language within caregiving. Contemporary sex offender literature highlights the requirement for support and management concurrently, that offenders' needs are important when working towards preventing future victimisation, that dynamic risk factors are important to consider and support (and that static risk factor analysis alone is misguided)—also that persons with sexual convictions can be both specialist and generalist offenders, which brings into the field elements of social exclusion, social capital, community inclusion, housing, employment, education, welfare, culture, etc. Walton and Hocken (43) conclude that interventions should assist people to “better respond to challenges in life” (p. 165). This contemporary holistic stance to work with persons with sexual convictions further illustrates the importance of care contexts and the norms and values therein.

### The Continuum of Men's Violence Against Women and Girls

The continuum of men's violence against women and girls was first coined by the feminist scholar Liz Kelly, who developed the theoretical framework to explore how women's experiences of men's violence are linked. Continuum thinking considers how “individual acts [exist] on a continuum [which] means seeing how they work *together*—in the context of a gender-unequal society—to produce particular effects on women's lives” [(44), p. 53]. The continuum approach supports a more nuanced understanding of VAWG that goes beyond othering those who enact the most violent gendered behaviour (e.g., sexual offending) and considers “gendered patterns of violence and experience[s]” that permeate everyday life for women [(45), p. 1]. Sexual violence has been suggested to be underpinned by the normative roles of heterosexual relationships, which are “imbued with the dominance-submission dynamic…where male aggression and female passivity are integral to the socially constructed roles” [(39), pp. 33–4].

These gendered norms are the “shared beliefs about what women or men do. They ascribe specific attributes, characteristics or roles to individuals because of their gender and are maintained by social approval or disapproval” [(46), p. 27]. Hegemonic masculinity is the culturally dominant form of masculinity within society, which “signals a position of cultural authority and leadership” and is often unevenly distributed amongst men [(47), p. 44]. The dominant form of masculinity promotes “attitudes and practices…that perpetuate gender inequality, involving both men's domination over women and the power of some men over other (often minority groups of) men” [(48), p. 113]. The roles of men and women which are often constructed as hierarchical and heteronormative whereby “[t]he nature of manhood is power, the nature of womanhood is subordination to power” underpins the continuum [(49), p. 20]. Therefore, the proposition is that gender roles and attitudes can become normalised in such a way which can support sexual violence and exists on a continuum of violence against women. The more common forms of sexual violence are often “defined by men as acceptable behaviour, for example seeing sexual harassment as 'a bit of fun' or 'only a joke', and they are less likely to be defined as crimes within the law” [(50), p. 49]. Thus, the continuum conceptualises incidents which fit outside the boundaries of criminality and considers the everyday encounters, which may be experienced as innocuous moments. This paper explores these gendered experiences during ethnographic fieldwork undertaken within an inpatient forensic mental health hospital in the UK.

The Me Too and Time's up movements, which have been argued to mark a major shift in gender equality (51), have reignited debates about sexual harassment (52). The notion that the individuals engaging VAWG are the deviant few, as represented by the media as rare instances, are argued to be unhelpful when gender inequality and VAWG is pervasive. Furthermore, attention often turns to individual women to become responsible for their own safety. Such an agentic approach is underpinned by neoliberalism which individualises problems and actions, rather than looking towards interventions that lay responsibility for problems, and resources to fix, within systems, institutions, agencies, etc. Thus, by “locating women as responsible for our safety, such campaigns also diminish the accountability not only of perpetrators, but of society and the state” [(46), p. 45]. Furthermore, such attention on women's safety work is a distraction from the wider issues linked to gender inequality, which “makes it harder to situate experiences of men's violence against women as a cause and consequence of gender inequality, rendering it instead an individual problem with an individual solution” [(46), p. 45].

A problematic victim-blaming narrative underpins much of the discussion around VAWG. It is proposed that the “structural and systemic nature of gender inequality, and the ways this plays out in everyday actions and interactions…[should be] a starting point for prevention” [(53), n.p.]. The continuum of VAWG provides a holistic, more nuanced view, which is suggested to more appropriately capture the experiences of women in everyday life.

### The Permeable Institution: Societal Issues

The notion that cultural and social norms (e.g., gender stereotypes and beliefs about masculinity and aggression) are supportive of violence against women is recognised (54). It is thus argued that cultural norms and practices, including inegalitarian attitudes towards women, infiltrate the exterior walls of institutions which aim to rehabilitate individuals who have engaged in gender-based violence. The permeability of institutions is often debated. Institutions have been conceptualised as a total institution in which a “barrier to social intercourse with the outside” exist [(55), pp. 15–16]. However, whilst it has been suggested that modern institutions have developed their permeability in various ways (e.g., short-stay patients, and for those in receipt of care: contact with those outside of the institution) (56), practices have been argued to be influenced by cultural, political, economic and legal factors (57). Thus, it is argued that political and socioeconomic elements from wider society permeate our porous institutions (58)—“the process of institutional infusion, in which an outside institution proffers attitudes, practices, and resources that individuals may draw on to shape their material and interpretive experiences within a host institution” (p. 175) is evidenced by Ellis (58), where religion is the example. This paper argues, through highlighting salient data from an institutional ethnography, that the social environment in forensic mental healthcare is influenced by heteronormative views of gender which are upheld by the macrosystem (i.e., at a societal level) and that such secure environments are unlikely to escape the wider oppressive system which reinforces patriarchal ideals both in subtle, but also at times, in overt ways.

## Methods

### Ethical Approval

A favourable ethical opinion was obtained from a NHS Research Ethics Committee, which specialised in qualitative research and the Mental Capacity Act (2005) (16/LO/0471). Residents' capacity to consent was assessed by the responsible clinician at the hospital, in accordance with Mental Capacity Act guidelines (59).

### Participants

The UK forensic mental health hospital cared for individuals with a history of offending or had presented with challenging behaviour and had been assessed as requiring care for their mental health. Residents were commonly detained under the Mental Health Act (1983/2007), with varying restrictions relating to their perceived risk to themselves and others. Informed consent was provided by the signing of consent forms from 14 staff (8 female, 6 male) and 9 male residents from two wards. Consent, however, was continually negotiated during fieldwork (60). A relational ethical approach was adopted to navigate the everyday ethical considerations when conducting research within a highly emotive environment. For self-care and fieldwork-reflection the researcher attended therapy during fieldwork, provided by the supervisory team, including a psychotherapist.

### Participant Observation

Overt participant observation was adopted in order to understand daily life at the hospital. The first author spent over 300 h within the inpatient forensic mental health hospital, observing and participating within daily life. Engagement in everyday conversations facilitates the observation of events and meaningful social intercourse (60), which is pertinent for the development of trust and the building of rapport (61). Ethnography is thus an embodied experience in which “evocative fieldnotes, vignettes, personal memories of taste, smell, conversations, music, angst and anger, joy and friendships, hard won familiarity and being marginal” [(62), p. 12] is central to the method.

Reflexivity is central to qualitative inquiry and is noted to “not simply [be] about researchers themselves, but also about how we are seen by the people we do research with and the power relations within these contexts” [(63), p. 451]. Thus, as noted earlier within this paper, the ways in which the ethnographer is perceived by the community is revealed through the interactions between the researcher and the members of the community, which also highlights cultural norms and accepted practices.

### The Female Ethnographer

It has been well documented that the gender of the ethnographer is integral to the ways in which the researcher is perceived and treated by the community. Gender not only shapes the encounters experienced by the ethnographer (64), but also reveals the role of gender within the community. For example, Ng (65) notes that exploring the inequalities and differences between the researcher and the community can lead to fruitful endeavours. Moreover, individuals within the community ultimately “transfer onto them [the researcher] definitions and images that belong to their own culture” [(66), pp. 67–68]. Female ethnographers often note their status as a female to be advantageous. For example, Haddow (67) found that gaining and maintaining access to an all-male community was promoted by their female status due to the community perceiving women as easier to get along with who don't present a threat to the male hierarchy. However, this was not without tension and Haddow (67) was indeed sexualised. Female ethnographers often write about their preparation for fieldwork and consider how their gender may present them with challenges, particularly when the community is all-male (68). The disclosure of abusive encounters in the field are presented as a warning to other junior or novice researchers so that they are prepared for fieldwork (69, 70). Fieldwork is a gendered process–both the process of creation of ethnographic data and the process of being in-the-field within patriarchal settings.

### Ethnographic Writing: Telling Tales of the Field

Ethnography aims to explore the other through participation within the daily lives of the community, however, such an endeavour is ultimately “experientially based” and adopts the approach of “I-witnessing” [(71), p. 53]. The community, then is perceived through the ethnographer's eyes and thus “ethnography is always partly autobiographical.” [(66), p. 65]. Ethnographic writing, or indeed their tales derived from their time conducting fieldwork, ultimately reflect the “personalised seeing, hearing, and experien[ces]” of the researcher [(72), p. 222]. Within this paper, ethnographic narratives are used as a powerful tool to “generate a sense of being there for the reader” [(73), p. 276], which are presented in the form of vignettes. Vignettes allow the reader “to sense some of the evocative power, embodiment, and understanding of life that comes through the concrete details of narrative” [(74), p. 9]. Thus, the narrative vignettes are deeply personal and include intimate details of the researcher, including their inner narrative and the emotions experienced. Ethnographic writing is produced “through which ethnographers render their experiences accessible to readers” [(73), p. 275], in which experiences or descriptions of the scene have been provided to evoke the reader to understand the embodied experience of the researcher. The narrative vignettes presented within this paper have been selected because they “aptly illustrate recurring patterns of behaviour or typical situations in that setting” [(75), p. 175] with a particular focus on gender roles.

### Data Analysis

Within ethnography, the data collection and analysis stages of research are often intertwined (76). For example, during fieldwork, the writing of the fieldnotes were found to heighten and focus the “…interpretive and analytic process” [(75), p. 100]. Emerging insights were added as additional headed sections following the write-up of observations in which accompanying theoretical codes or insights were noted. The research adopted a constructionist ontological position which appreciates that social phenomena are constructed (77). An emic approach to knowledge was adopted which aims to understand the local interpretation (78) of community life at the hospital in which “components of a cultural system from the perspective of the group being studied” is considered [(79), p. 16]. The aim then was to understand the perspectives of those who work and reside at the hospital, adopting an inductive approach.

### Strengths and Limitations

This paper considers gender role stereotypes within the inpatient forensic mental health context, which has synergies with other important work in this field (80). A limitation of this research is that the demographic details, such as, age and ethnicity, were not collected within this research; meaning that the sample cannot be situated (81). Furthermore, an understanding of the importance of these influences (and biases/hierarchies) would have been advantageous for the exploration of intersectionality. For example, hegemonic masculinity not only reinforces power structures relating to gender, but also sexuality (48) and race (82). A commitment to such notions of masculinity serves to marginalise those who do not fit the social norms of masculinity (83) and this commitment may indeed have been performed within the institutional community. Racial abuse experienced by staff and those in receipt of care is prevalent within the forensic mental health context (4), thus intersectionality would undoubtedly be relevant to the arguments presented within this paper.

## Results and Vignette Structure

This paper focuses on data from two male wards in order to critically explore our data pertaining to gender roles, masculinity, and heteronormativity. Our central argument is that the intertwining and interdependent cultural and custodial elements of forensic healthcare environments are integral and influential to care, culture, and conduct within such institutions–including concerning misogynistic everyday talk and the continuum of men's violence against women therein. A series of narrative vignettes are presented, which illuminate the gendered environment and the upholding of normative gender roles, some of which include the subjugation of women. Details not pertinent to the analysis have been changed to maintain confidentiality and pseudonyms have been used throughout. The five vignettes are presented, then discussion occurs after the presentation of data. This string of continuous ethnographic data has been selected in order for the reader to experience the fieldwork setting uninterrupted. The five vignettes are arranged in escalation order, from more commonly experienced to less commonly experienced, to demonstrate the continuum.

### Vignette 1: “That's a Bit Girly”

We were on a community visit to a local bowling alley. I sat with the occupational staff, Jessica and Nicole, on a tall stool, which overlooked the lanes being occupied by the residents. It was nearing the end of the session; the lights turned off above the isle lanes, indicating that time was up. The residents gathered in the foyer. We stepped outside. The taxi wasn't waiting as it usually was. Nicole walked away a little to phone the taxi company and indicated that'll it'll probably be here shortly. Toby sat down on the grass with Jessica, I joined them. It was a nice day as we basked in the sunshine. It was Summer, the grass was a little overgrown with bunches of daisies. Toby started to make daisy chains. I started looking for a daisy that had a long and sturdy-looking stem. I found one, picked it, and began handing it over to Toby commenting: “This looks like a good one.” Nicole started walking back over saying the taxi was just coming, she looked up the road. I turned to see the taxi pulling in. I stood up. Nicole looked down at what Toby was doing, she frowned and scoffed: “That's a bit girly isn't it?! Daisy chains! Come on, the taxi is here.”

### Vignette 2: “You Want Them Pretty”

This vignette is an observed moment at the hospital between a male staff member, Mark, and a male resident, Jacob. This interaction took place after seven months in the field.

I was invited by Mark to accompany him on leave with Jacob, a resident on the ward. Jacob was approximately 20 years his junior. We crossed the road and stood in the doorway of an abandoned shop. Jacob lit his cigarette. He was looking down at his phone and swiping. After taking a long drag on his cigarette, he turned the screen, showing it to Mark and continued to hold his breath before exhaling: “Look at her, what do you think?” Jacob asked. “Yeah, she's nice, she's pretty,” Mark responded. Jacob asked: “Yeah, you think?.” “You want them pretty, but not too intelligent! That was the problem with my ex, she was really pretty and intelligent, it caused us problems,” Mark responded. “Oh yeah?” Jacob replied with a slight chuckle. “Yeah mate,” Mark responded raising his eyebrows as a signal of the certainty of his statement. I stood a little distance away from Mark and Jacob, remaining silent. I felt ignored and overlooked as a bystander to this inappropriate kind of male locker room talk.

I reflected at the time: Jacob was the newest member to join the resident community on one of the male wards. The day before I overheard a conversation between two members of female staff. They were discussing Jacob and his comments to female staff. He kept referring to them as “woman.” They were annoyed and talked about how they had been challenging him on this, but he seemed to find it amusing. They said that this was becoming problematic and agreed that they needed to keep challenging him on this, it wasn't okay. I'd noticed that before this, Jacob had called me woman and I challenged him on this and asked him not to call me “woman” –I had a name. Jacob merely laughed in response.

### Vignette 3: “Her Name Is Slopey Shoulders”

I was on one of the male wards, it was mid-morning. I was by the ward kitchen door, which had a glass panel, I looked to see if the cooking session with occupational therapy had started yet. I heard someone coming down the corridor, I glanced over to see if it was Jarred who usually cooks during this time. I could see it was a female member of staff who I hadn't met before. I began introducing myself as Daniel appeared, he overheard the conversation. He beckoned loudly “Don't you know her name, it's slopey shoulders?” (Slopey shoulders is used to describe someone who is devoid of responsibility. They are viewed as someone who does not carry any weight of responsibility on their shoulders). I had prepared myself for a meeting such as this. Daniel had started to call me this name and it was starting to get annoying. I planned to challenge him, but only when it was staff that was present. I didn't want to divert the focus from my observations or interactions with the community. The new member of staff was stood facing me, Daniel was behind her a few feet away. I looked at Daniel, shook my head and replied “No, that's not my name.” I turned to face the female member of staff and smiled: “So, my name is Emma, I'm a researcher….” Daniel interrupted again “Yes, it is, it's slopey shoulders!.” He'd walked closer to where we were stood. I turned and remarked “No, it's not.” The female member of staff began walking away and said, “Oh leave it you two.” She walked back down the corridor, away from us, she was gone. Daniel stepped towards me and moved his body slowly and purposefully, standing tall with his shoulders back, like he was squaring up to me, almost ready for a fight. “That's what we call you, slopey shoulders,” he was towering over me now, his body positioned in a threatening manner but his speech contrasted this? he spoke as if this was all a joke, just banter. My mind turned to the security camera–I knew we were in shot. I looked up at Daniel, “please don't call me that, my name is Emma.” I felt the vibration of the security alarm on my lower back and the sound of the alarm, it was jarring. I turned to look to see what the message was on the display. I could see Daniel had begun walking away quickly whilst glancing at his alarm. He was gone.

### Vignette 4: “You Need to Be Careful, You're Pretty and Young”

It was the afternoon, I headed over to one of the male wards. I opened the first door with the heavy set of keys and placed these back in my pouch that was secured to my belt. I hovered by the Perspex window which provided a view into the staff office from the security airlock. A staff member clocked me. I smiled and mouthed “hi.” They were talking to another member of staff and moved over towards the door release situated in the office. I heard the click of the door being released. I opened the door and entered the ward. Joe, a resident, was across the room, near to the kitchen. He smiled and nodded, I smiled back. I noticed there were a couple of tabloid papers on one of the dining room tables, they looked new, perhaps today's paper. One of them had been left open, on a page with a large photograph of a woman in underwear.

Andrew was stood near the staff room door: “Oi, come here for a moment” He nodded his head to the side to invite me over towards him. “I need to have a chat with you” he beckoned across the ward. I walked over to him expecting us to have the chat on the main ward. He turned around to face one of the side room doors. He tugged at the leather rope to pull his set of keys out of his trouser pockets and proceeded to swing them upwards, he caught them with his hand, clashing the set of keys together as he closed his fist. He unlocked the door and headed into one of the side rooms; it was a small room. He stepped in and turned around to address me, leaving only a foot between us. Andrew is taller than me, which felt very noticeable now we were stood in close proximity. I had to tilt my head back to look up at him. My mind was racing: What did he need to talk to me about? He started talking about James, one of the residents that I knew well. “James has a history with young women.” I was nodding my head to indicate my knowledge of this. I did know about his previous offence. He continued: “You need to be careful, you're pretty and young.” I understood what Andrew was saying but I felt uncomfortable with his comments about my looks and my age. I also knew that I wasn't quite as “young” as he seemed to think I was. He said “okay?” to indicate that we were done. We left the side room to join the ward.

I reflected at the time: I understood James' history. I didn't consider my appearance or perceived young age as risk factors. I reflected on this interaction during a clinical supervision session. We discussed how my attributes were positioned as risky within the hospital environment. It was almost as if my looks and apparent young age were being pathologised–*I* was somehow the risk? it was my fault, and my responsibility to manage this. This also wasn't the first time I had been called young. Another time I was on the same male ward with three male residents. One of the men brought up the topic of tattoos and asked if I had any. Another male resident commented, before I had the chance to respond myself, that of course I didn't, I was far too young to even have a tattoo. I was 29 at the time.

### Vignette 5: “He Had His Hands Down His Trousers”

It was the afternoon, after lunch. A few of the residents were sat in the communal area, watching the TV on the sofas. The music channel was on. One of Ed Sheeran's songs was playing “kissed her on the neck and then I took her by the hand…my pretty little Galway girl.” I sat down next to Johnathan, who I knew quite well. We exchanged pleasantries and chatted about the music session that happened earlier that day. We sat in silence for a few moments. I glanced at the TV and then looked around the ward to see a staff member coming down one of the corridors from the bedrooms. They headed to the office. I soon realised that Johnathan had his hands down his trousers–he was pleasuring himself! I stared back at the TV, which meant I could face away from him. I thought for a moment. My mind was racing. Should I say something? Had anyone noticed? I searched around with my eyes trying to not move my head from the direction of the TV. It didn't seem like anyone had, the residents were still watching TV, one of them was laying on the sofa, not facing us. I looked straight ahead at the clock so I could see out the corner of my eye–just to cheque. Did he still have his hands down his trousers? Were his hands still moving? They were. I could see his head was back and his eyes were closed. I asked myself: Why am I still sat here? I didn't want to draw any attention to it, is that why I was still there? I decided that I needed to leave. I slowly stood up and headed to the staff base, not looking back.

When I entered the staff base, there were a few female staff members. I stood, probably looking quite shocked, as one of the staff members looked at me. Their attention was suddenly on me. I explained what happened. There seemed to be some shock. I was asked if I was okay. I indicated that I was.

I reflected afterwards: This felt like the most attention that I'd received from the staff. They were usually busy. I understood. But this felt different. The female staff took the time to cheque in with me and run through what had happened. It felt caring. We didn't know each other well. It clearly felt important that we explored what had happened. I felt numb. This seemed in contrast to the staff's reaction.

## Discussion

The cultural and custodial elements of forensic healthcare environments are integral to care, culture, and conduct. The social environment is influenced by contemporary social issues as the boundaries of such institutions are porous. Misogynistic everyday talk and the continuum of men's violence against women are thus important to explore, which have implications for the therapeutic milieu. The researcher experienced sexual harassment and violence during fieldwork (e.g., vignette 5) and such experiences are prevalent in modern mental healthcare. Indeed the Care Quality Commission (84) has called for national guidance to improve sexual safety on mental health wards, following reports of sexual harassment and violence in mental healthcare in the UK. Such reports have been filed by staff and individuals in receipt of care, which include both staff and those in receipt of care as perpetrators from a range of settings, including acute adult wards, forensic units, and child and adolescent units. The experience of the researcher, then, is not uncommon and perhaps, unsurprisingly, such experiences have been shared by other ethnographers, albeit in different contexts. Writing of the sexual assault that Grenier [(69), p. 8] experienced, they conclude that “this type of incident can occur even in the course of our everyday lives” and it's important to consider that such moments only serve “to highlight a reality shared by numerous female ethnographers.”

As noted earlier, the ways in which individuals interact with the ethnographer reveals much about cultural norms and practices, and ethnographers are often sexually positioned by participants during fieldwork (63, 67, 69). The experience of the researcher within this study highlights how she was viewed by those encountered at the institution and, importantly, reveals the power relations embedded within the context. This paper explores everyday social interactions and the embodied experience of the female ethnographer within a male-dominated forensic setting through a series of ethnographic observations evidencing a series of encounters rooted in patriarchal views of women that underpin gendered violence.

The forensic mental health environment has been described as a “male space … [which] promote[s] gendered inequality” [(85), p. 15]. Salient issues evidenced within the ethnographic work include heteronormative gender roles, hegemonic masculinity, and misogynism. Interactions between staff and those in receipt of care underpin therapeutic work and within forensic mental health, a corrective behavioural approach is commonly adopted (23). Within the institution, this approach was adopted to uphold gendered norms (e.g., vignette 1) when a resident was discouraged from engaging in “girly” activities (e.g., making a daisy chain). As noted previously within this paper, the corrective approach aims to promote socially acceptable behaviour (25), and in this instance, the behaviour that was being corrected represented the upholding of heteronormative gender roles. Hegemonic masculine attitudes and practices are upheld by both men and women, but such rigid ideas of masculinity can harm men (48) or serve to constrain men (47). In vignette 1 Toby was discouraged from engaging in the supposed feminine activity which upholds the values attributed to hegemonic masculinity, however in the context of mental health rehabilitative work, such an activity could be viewed as therapeutic. Thus, the upholding of rigid gender norms and the teaching of these were values, that permeated the secure setting, directed the content of the regime.

Hierarchical and heteronormative roles in which the male is dominant and the female undertakes a submissive position within relationships underpins the continuum of Violence Against Women and Girls (VAWG) (49). Furthermore, these socially constructed roles create the foundation for coercion as normative (39). The “lad's talk” presented within vignette 2 where Mark shares with Jacob that “[y]ou want them [women] pretty, but not too intelligent!” is underpinned by rigid heteronormative gender roles. Whilst it is recognised that the “relationship between gender and violence is complex…in many societies, women are viewed as subordinate to men and have a lower social status, allowing men control over, and greater decision-making power than women” [(86), p. 81]. Mark shares his views of the desirable attributes of women, in which she is “not too intelligent,” perpetuates this notion that men should be dominant in heteronormative interpersonal relationships (i.e., notions linked to hegemonic masculinity). The adherence to rigid gender roles increases the “likelihood of violence against women” [(87), p. 279] and reproductions of restrictive notions of masculinity is a “key aspect of complicity of violence against women” [(88), p. 11]. Forensic mental health settings should examine their gendered values and wherein steps towards gender equality might be forged.

Hegemonic masculinity is now problematised within the sexual offending rehabilitative context. The GLM approach to offender rehabilitation aims to assist individuals to achieve their goals through appropriate methods in order to manage their risk of reoffending. Thus, clinical work would aim to challenge the views of those who use inappropriate methods of obtaining primary goods, for example, seeking intimacy through violence or controlling behaviour, or indeed aim to obtain dominance over another individual through sexual violence. Ward and Brown (37) further explain how “[s]ome of these [risk] factors are causally related to offending behaviour in a fundamental way (for example, antisocial attitudes).” It is argued that Mark's comments exhibit an antisocial attitude towards women and such views can be associated with sexual offending, which have the potential to reinforce existent “cognitive distortions” for individuals. It is claimed that these comments can normalise already problematic views of heterosexual relationships which may be described by clinicians as cognitive distortions. The sharing of information through “innocuous personal stories” has been found to assist in the building of trust between forensic patients and staff [(12), p. 755] and whilst interpersonal relationships have been suggested to be the first step in rehabilitation/recovery, such comments that undermine women are out of the scope of appropriate conversations. From a rehabilitative perspective then, the institution would aim to challenge such distortions associated with the degrading of women (e.g., that women should not be intelligent).

In vignette 2, Mark, whilst relating to Jacob on an interpersonal level by sharing his views of heteronormative dating, is also adopting a paternalistic approach and teaches Jacob what is desirable when searching for a female partner. Research conducted within the forensic setting found that “[t]he term ‘lad's talk’ described an informal feature of life, when common interests replaced difference in upholding masculine values…with sport and sex acting as metaphors of masculinity” [(89), p. 177]. The researchers found that male nurses adopted an othering approach when referring to those in receipt of care, except, interestingly, when referring to themselves as men, thus indicating the inter-group relatedness of being male. As noted earlier, the building of relationships is fundamental for promoting relationship-enabling care, which lays the foundation for the teaching of acceptable behaviour (25). However, it's important to consider how the upholding of hegemonic masculinity, which are situated at a societal level (the macro) reinforces male dominance and the oppression of women, even in everyday talk, which may seem innocuous if it's considered to be removed from or not connected to the deviant few (e.g., those who engage in sexual violence). It is therefore argued that the “extent to which male dominance and the oppression of women is embedded in the ways that we see the world and conduct ourselves in it means that we cannot simply divorce ourselves from that system if we wish to do so” [(90), p. 46]. Overt staff attitudes towards women and rigid gender roles were evidenced within everyday talk, which not only serve to undermine clinical-rehabilitative work, but also highlights the embedded and normalised nature of VAWG.

It is argued that oppressive attitudes of women permeate institutions and are evidenced in everyday encounters, which has implications across the criminal justice system. For example, it has been argued that the “police and courts operate within the context of a society shaped by patriarchy, … [which are] still characterised by high-levels of victim-blaming and rape-supportive beliefs” [(91), p. 267]. Forensic mental healthcare and rehabilitative contexts too are situated within this system. The ethnographer's perceived level of “prettiness” and “youth” was viewed as a risk factor for one of the male residents, and the intervention to manage this risk was to be managed by the ethnographer by “being careful.” This approach is underpinned by a victim-blaming rhetoric. In this sense, the personal is indeed political–the researcher has been advised to navigate the risks associated with her perceived attributes, however, it's important to recognise how this individual experience is understood at “multilevel contexts, [including] institutional as well as socio-historical and geopolitical” (92). Thus, the victim-blaming narrative upholds the notion that women should implement safety work, which is located at the individual level, rendering the victim as responsible—such a view, as discussed earlier in this paper, is problematic.

Moving on to vignettes 3 and 4, whilst name-calling may seem innocuous, such discriminatory behaviours “violate dignity to create a hostile environment” and can be enacted through “derogatory comments that undermine…identity” (92). The researcher experienced bullying and threatening behaviour both verbally (e.g., name-calling) and physically (e.g., one of the members of staff “squared up” and towered over the female researcher). Discrimination-compliant culture “that perpetuates or ignores acts of everyday sexism, racist microaggressions, homophobic and other workplace “banter” (92). was evident within the institution and, within this research, is linked to hegemonic masculinity.

Further, it is proposed that:

Failing to recognise and address the ways in which gendered inequalities pervade all areas of social life, including our own, heightens the risk that they will be reproduced unchallenged within the field of engaging men too [(90), p. 46].

It is important to consider the everyday conversations that occur between staff and patients within the offending context and understand how such social interactions may represent a wider inegalitarian rhetoric, which may serve to normalise VAWG and, from a clinical-rehabilitative perspective, criminogenic attitudes and behaviour. Thus, whilst mutual engagement may positively influence the staff-patient relationship and promote recovery, gendered attitudes held by some staff serve to undermine therapeutic practices and rehabilitative work.

## Conclusions and Implications

Practices, including everyday talk, which promote the subordination of women are supportive of violence against women (86) and approaches which aim to challenge such views underpin violence prevention strategies (54). Thus, violence prevention strategies aim to promote gender equality by “challenging stereotypes that give men power over women” [(86), p. 80]. The notion that men can engage in work to challenge the inequalities experienced by women is an area which has been importantly receiving much attention. For example, Jewkes et al. (93) argue that men shouldn't only be viewed as perpetrators of violence but as allies in the prevention of Violence Against Women and Girls (VAWG). Furthermore, targeted approaches which focus on particular men (e.g., those convicted of crimes relating to violence against women) limits the impacts of interventions evading wider social transformation (48). Thus, a continuum approach to VAWG allows for a broader understanding of the structural and systemic issues experienced by women and aims to look beyond an othering approach in which certain men are seen as the problem (50). Such defensive thinking contributes to a disengagement with a more nuanced understanding of VAWG, which serve to undermine experiences of gendered patterns of violence that “permeate everyday life for women” [(45), p. 1], which may be overt or indeed subtle.

Within this research, the therapeutic milieu was influenced by hierarchical heteronormative gender roles, violent language in forensic settings, victim-blaming and misogynistic attitudes and practice, on the care for, and rehabilitation of, patients. Hegemonic masculinity was observed to reinforce gender order in various ways. For example, by constraining activities considered outside of masculine norms and reinforcing notions that men should be in a position of power and women should undertake a submissive role—particularly within heterosexual relationships. Hegemonic masculinity is not informed by fixed ideas of gender roles but is fluid. Ideas of masculinity are reinforced through social practices (47) and thus, there are continual opportunities for growth and change. Divergent forms of masculinity exist and forms that challenge existing power structures between men and women are being realised—however, not without its challenges (88). Further, within the custodial environment, masculinity presents particular challenges, with exaggerated masculinity viewed as a coping or survival strategy. However, within the Therapeutic Community model, principles of collective responsibility, empowerment, and citizenship underpin community life (94). Such an environment is incongruent with hegemonic masculinity and community members experience the dismantling of these conceptions of masculinity through therapy and community living (95). The Therapeutic Community model creates an environment in which everyday constructions of hypermasculinity can be challenged and new constructions of hegemonic masculinity can be embraced and supported, by all community members including staff. Our previous paper advocated for the Therapeutic Community model within forensic environments in response to the challenging interpersonal environment in which racism, violence and bullying was observed by the ethnographer (4). Once again, this model is advocated for, particularly within the context where individuals are undertaking rehabilitative work related to sexual offending, so that everyday social encounters can be underpinned by an egalitarian ethic, one that challenges the gender inequalities which pervade social life and indeed our institutions, and contribute to the continuum and the continuation of VAWG.

## Data Availability Statement

The original contributions presented in the study are included in the article/supplementary material, further inquiries can be directed to the corresponding author.

## Ethics Statement

The studies involving human participants were reviewed and approved by NHS Research Ethics Committee. The patients/participants provided their written informed consent to participate in this study.

## Author Contributions

The ethnography was conducted by EJ as part of her doctoral studies at the University of Nottingham. MJ was a supervisor on this doctoral work. This paper has been prepared by EJ and MJ. Both authors contributed to the article and approved the submitted version.

## Funding

This original research was funded institutionally as part of a larger Arts and Humanities Research Council (AHRC) Connected Communities Programme Grant (AH/K003364/1), Creative Practice as Mutual Recovery led by Professor Paul Crawford. The funders had no role in study design, data collection and analysis, decision to publish, or preparation of the manuscript. Open access publication fees are supplied by Newcastle University.

## Conflict of Interest

The authors declare that the research was conducted in the absence of any commercial or financial relationships that could be construed as a potential conflict of interest.

## Publisher's Note

All claims expressed in this article are solely those of the authors and do not necessarily represent those of their affiliated organizations, or those of the publisher, the editors and the reviewers. Any product that may be evaluated in this article, or claim that may be made by its manufacturer, is not guaranteed or endorsed by the publisher.
